# Measures for interoperability of phenotypic data: minimum information requirements and formatting

**DOI:** 10.1186/s13007-016-0144-4

**Published:** 2016-11-09

**Authors:** Hanna Ćwiek-Kupczyńska, Thomas Altmann, Daniel Arend, Elizabeth Arnaud, Dijun Chen, Guillaume Cornut, Fabio Fiorani, Wojciech Frohmberg, Astrid Junker, Christian Klukas, Matthias Lange, Cezary Mazurek, Anahita Nafissi, Pascal Neveu, Jan van Oeveren, Cyril Pommier, Hendrik Poorter, Philippe Rocca-Serra, Susanna-Assunta Sansone, Uwe Scholz, Marco van Schriek, Ümit Seren, Björn Usadel, Stephan Weise, Paul Kersey, Paweł Krajewski

**Affiliations:** 1 Institute of Plant Genetics, Polish Academy of Sciences, ul. Strzeszyńska 34, 60-479 Poznań, Poland; 2 Leibniz Institute of Plant Genetics and Crop Plant Research (IPK), OT Gatersleben, Corrensstraße 3, 06466 Stadt Seeland, Germany; 3 Bioversity International, parc Scientifique Agropolis II, 34397 Montpellier Cedex 5, France; 4 Institute for Biochemistry and Biology, University of Potsdam, 14476 Potsdam, Germany; 5 INRA, UR1164 URGI - Research Unit in Genomics-Info, INRA de Versailles-Grignon, Route de Saint-Cyr, 78026 Versailles, France; 6 Forschungszentrum Jülich GmbH, IBG-2 Plant Sciences, 52425 Jülich, Germany; 7 Institute of Computing Science, Poznań University of Technology, ul. Piotrowo 3a, 60-479 Poznań, Poland; 8 LemnaTec GmbH, Pascalstraße 59, 52076 Aachen, Germany; 9 Poznań Supercomputing and Networking Center Affiliated to the Institute of Bioorganic Chemistry, Polish Academy of Sciences, ul. Jana Pawła II 10, 61-139 Poznań, Poland; 10 UMR MISTEA, INRA SupAgro, Place Pierre Viala 2, 34060 Montpellier, France; 11 Keygene N.V., Agro Business Park 90, 6708 PW Wageningen, The Netherlands; 12 Oxford e-Research Centre, University of Oxford, 7 Keble Road, Oxford, OX1 3QG UK; 13 Gregor Mendel Institute, Austrian Academy of Sciences, 1030 Vienna, Austria; 14 Institute of Biology I, BioSC, RWTH Aachen, Worringer Weg 3, Aachen, Germany; 15 The European Molecular Biology Laboratory–The European Bioinformatics Institute, Wellcome Trust Genome Campus, Hinxton, Cambridgeshire CB10 1SD UK

**Keywords:** Data standardisation and formatting, Experimental metadata, Minimum information recommendations, Plant phenotyping, Experiment description

## Abstract

**Background:**

Plant phenotypic data shrouds a wealth of information which, when accurately analysed and linked to other data types, brings to light the knowledge about the mechanisms of life. As phenotyping is a field of research comprising manifold, diverse and time-consuming experiments, the findings can be fostered by reusing and combining existing datasets. Their correct interpretation, and thus replicability, comparability and interoperability, is possible provided that the collected observations are equipped with an adequate set of metadata. So far there have been no common standards governing phenotypic data description, which hampered data exchange and reuse.

**Results:**

In this paper we propose the guidelines for proper handling of the information about plant phenotyping experiments, in terms of both the recommended content of the description and its formatting. We provide a document called “Minimum Information About a Plant Phenotyping Experiment”, which specifies what information about each experiment should be given, and a Phenotyping Configuration for the ISA-Tab format, which allows to practically organise this information within a dataset. We provide examples of ISA-Tab-formatted phenotypic data, and a general description of a few systems where the recommendations have been implemented.

**Conclusions:**

Acceptance of the rules described in this paper by the plant phenotyping community will help to achieve findable, accessible, interoperable and reusable data.

**Electronic supplementary material:**

The online version of this article (doi:10.1186/s13007-016-0144-4) contains supplementary material, which is available to authorized users.

## Background

Plant research routinely uses a multitude of techniques and increasingly advanced types of analyses. Scientists delve into a wide range of characteristics manifesting themselves at all levels of plant structure and over their life cycles. The resulting data encompassing genome, epigenome, transcriptome, proteome, and metabolome, and the expression of all other traits (economically or otherwise important) should be integrated to provide a better understanding of the plant systems. The quality and cost of such integration is, however, critically conditioned by the interoperability of the underlying data, i.e., by the availability of adequate metadata describing datasets, and the compatibility of the metadata and data contributed by different scientists, both in terms of the content and the structure. Meanwhile, some plant research fields, especially phenotyping, still lack proper standardization policies to facilitate effective data exchange and integration [1].

Phenotyping is a very wide and heterogeneous research field. It analyses both static quantities and dynamic processes. Sensitivity of the phenotypic observations to environmental conditions (in the sense of the genotype-by-environment interaction, G × E) requires scrupulous data handling for the acquired signal to be optimally preserved and persisted in databases to deliver most substantial scientific value. Meanwhile, differing amounts of metadata about experiment set-ups, lots of different trait names and their synonyms, and diverse rating scales are used (e.g. [2, 3]), leading to ambiguity and inconsistency of phenotypic data description. Hence, both correct integration and interpretation of phenotyping experiments is hampered. Actions undertaken so far for phenotypic data have either been project-specific (DROPS [4]), platform-specific (PODD [5, 6]; Phenome FPPN [7]), or database-specific (MaizeGDB [8], Triticeae Toolbox [9], Phenopsis DB [10], GnpIS-Ephesis [11]). The lack of common standards of plant phenotyping experiments’ description, both in terms of its content and the format, hampers the correct usage and re-usage of phenotypic data.

A proper description of experimental metadata is a key to the correct interpretation of the outcome. In many research domains there have been initiatives aiming at provisioning of recommendations for the set of metadata needed to describe experimental results of particular biological assays. Most of them have resulted in a formulation of a “Minimum Information” or a similar “checklist” document, containing assay-specific recommendations. For example, the Genomic Standards Initiative formulated requirements for reporting sequences of nucleotides (MIxS [12]). The Microarray Gene Expression Database Group suggested the requirements for the description of transcriptomic data (MIAME/Plant [13]). The Proteomics Standards Initiative published a corresponding set of recommendations for protein data (MIAPE [14]). Finally, the Metabolomics Standards Initiative provided rules concerning metabolomic observations (CIMR [15–17]) that were recently considered as a basis for more formal standardization by Rocca-Serra et al. [18]. These documents agree—in principle—on how to describe the experimental material and the treatments applied to it. A similar approach seems advisable to provide metadata recommendations for plant phenotypic data.

As far as data formatting is concerned, for most data types the existing policies are database-specific. Formats that gained wider acceptance are MAGE-TAB [19], a text, tabular format required by the ArrayExpress database [20], storing gene expression data, and PRIDE XML or mzIdentML, required by the PRIDE database [21] for proteomics data. The ISA-Tab format [22] has been developed to address descriptions for many types of experiments and assays. Its flexibility and focus on the experimental metadata, clearly separated from the data itself, make ISA-Tab a generic solution, now used by a number of research communities [23], with a potential to constitute a general experimental metadata description standard, also for phenotypes.

In this paper, we report the measures taken to standardize the description of plant phenotypic data. We present solutions that are a concrete implementation of the opinions expressed recently by many partners of two European infrastructural projects, transPLANT (Trans-national Infrastructure for Plant Genomic Science [24]) and EPPN (European Plant Phenotyping Network [25]) in [1]. The solutions are generic and intended to systematize the way of describing all types of phenotypic data independently of the particular local requirements of a project or database, and thus aim for a better interoperability. At the same time, our propositions take into account the achievements of other omics- and phenotype-oriented initiatives, including the above mentioned.

We provide a document called “Minimum Information About a Plant Phenotyping Experiment” (MIAPPE). It constitutes a list of attributes that, based on our experience, are necessary for a useful description of a plant phenotyping experiment and understanding of the data obtained in it. In particular, it comprises recommendations given by Poorter et al. [26] and Hannemann et al. [27] about the documentation of environmental parameters during the experiment, which is a crucial aspect in a G × E-aware phenotype analysis.

As to the way of formatting the metadata, we propose using the above-mentioned ISA-Tab structure for experimental metadata collection and exchange. We show that ISA-Tab, thanks to its generality and flexibility, can handle multitude of phenotyping experiment types and designs. Also, due to its application by several projects and platforms (see [23]), it promotes compatibility of our propositions with those concerning other data types.

Interoperability cannot take place without semantic annotation of the data with respect to the publicly available, controlled vocabularies and ontologies, which provide a community vetted language. This must be done at least for properly identified pivot objects, or key resources, i.e. the elements of a given dataset that allow its integration with other datasets. While the use of particular ontologies is not our main topic, we provide some recommendations in this area. Importantly, all annotations can be conveyed by the ISA-Tab formatted files.

Finally, we present example datasets constructed according to the methods described. Technical aspects of dataset construction and data annotation using recommended ontologies are not covered in this paper; we give some general remarks and refer to existing tools designed for these tasks. We present a few examples of systems where the recommendations have been (or are being) implemented and tested. Some of them are based on own tools and databases, others make use of publicly available utilities provided by the developers of ISA-Tab format [28]. They demonstrate some use cases where the approach described in this paper proved suitable.

## Results

### Minimum Information About a Plant Phenotyping Experiment (MIAPPE)

The Minimum Information About a Plant Phenotyping Experiment is a list of attributes that we recommend for the description of phenotypic observations. It contains the properties that should be provided (by a person or system depositing the data) alongside experimental results to ensure easy and correct interpretation, assessment, review and reproducibility.

To create the recommendations contained in MIAPPE, we took into account previously created Minimum Information documents for various branches of biological research: MIxS for sequences, MIAME/Plant for transcriptomics, MIAPE for proteomics, and CIMR for metabolomics, and have re-used their attribute definition where appropriate. In many cases, where several standards touch upon the same data type (e.g. general metadata, timing and location, treatments), they do so in a compatible fashion, making it straightforward to adopt existing recommendations. Yet, for a number of data types we had to make a choice which approach to adopt. Finally, some information had not been described in the existing documents, which called for provision of such a description from scratch.

The MIAPPE checklist consists of attributes that can be classified within the following sections:

- General metadata,
- Timing and location,
- Biosource,
- Environment,
- Treatments,
- Experimental design,
- Sample collection, processing, management,
- Observed variables.

Each section aggregates attributes detailing specific aspects of an experiment that are important to note, where applicable. The full list of MIAPPE attributes, their origins, and the reasons behind their selection, are given in Table 1. Below, we justify the presence of particular MIAPPE sections.

**Table 1:** Minimum Information About a Plant Phenotyping Experiment (MIAPPE)

Checklist section	Attributes	Source list/biosharing ID/reference	Recommended ontologies
General metadata	Unique identifier* Title* Description* Submission date Public release date Publications Laboratory address and contact details	ISA reporting standard [65]	OBI, Ontology for Biomedical Investigations [66] CRO, Crop Research Ontology [35]
Timing and location	Timing: Start of experiment (date/hour)* Duration (hours/days/months/years)* Experiment location: Geographic location* Latitude and longitude Altitude Inclination and aspect Habitat	Poorter et al. [26] Morrison et al. [17] CIMR [67]: Environmental Analysis Context	OBI, Ontology for Biomedical Investigations [66] GAZ, Gazetteer [68]
Biosource	Organism (taxon)* Infraspecific_name* Infraspecific_rank Common name Genotype Organism age Life stage Seed preparation: Seed source* Pretreatments Conservation conditions	MIxS Plant-associated environmetal package [69] Yilmaz et al. [12] FAO/Bioversity Multi-Crop Passport Descriptors V.2 (MCPD V.2) [30]	UNIPROT Taxonomy [70] NCBI Taxonomy [71]
Environment	Growth facility* (growth chamber, GC/greenhouse, GH/open top chamber, OTC/experimental garden/experimental field)	Poorter et al. [26] Hanneman et al. [27]	XEO, XEML Environment Ontology [36] ENVO, Ontology of environmental features and habitats [72] Crop Research Ontology [35]
	Aerial conditions* CO_2_ For GC and GH: Controlled/uncontrolled Average CO_2_ during the light and dark period (µmol mol^−1^) Air humidity (moisture)* Average VPDair during the light period (kPa) or average humidity during the light period (%) Average VPDair during the night (kPa) or average humidity during the night (%) Daily photon flux (light intensity)* Average daily integrated PPFD measured at plant or canopy level (mol m^−2^ day^−1^) Average length of the light period (h) For GC: Light intensity (µmol m^−2^ s^−1^) Range in peak light intensity (µmol m^−2^ s^−1^) For GH and OTC: Fraction of outside light intercepted by growth facility components and surrounding structures Light quality: For GC and GH: Type of lamps used R/FR ratio (mol mol^−1^) Temperature (°C)* Average day temperature Average night temperature Change over the course of experiment		
	Rooting conditions* Rooting medium*: aeroponics/hydroponics (water-based, solid-media based)/soil type (sand, peat, clay, mixed, …) For greenhouse: Container type* Volume (L)* Height Other dimensions* Number of plants per container* For field: Plot size* Sowing density* pH* Frequency and volume of replenishment or addition Soil parameters: Soil penetration strength (Pa m^−2^) Water retention capacity (g g^−1^ dry weight) Organic matter content (%) Porosity (%) Rooting medium temperature		
	Nutrients For hydroponics: Composition* Concentration For soil: Extractable N content per unit ground area before fertiliser added* Type and amount of fertiliser added per container or m^2^* Concentration of P and other nutrients before start of the experiment Extractable N content per unit ground area at the end of the experiment		
	Watering Irrigation type: irrigation from top/bottom/drip irrigation* Volume (L) and frequency of water added per container or m^2^* For soil: Range in water potential (MPa)		
	Salinity Concentration of Na, Cl and Mg in the water used for irrigation For soils and hydroponics: Electrical conductivity (dS m^−1^)		
	Aquatic environment If sample was submerged and emerged Depth Time Water temperature Tidal phase		
	Biotic environment Description of interacting organism (pathogens, mutualists, herbivores, endophytes, etc.)		
Treatments	Seasonal environment Air temperature regime Soil temperature regime Antibiotic regime Chemical administration Disease status Fertilizer regime Fungicide regime Gaseous regime Gravity Growth hormone regime Herbicide regime Mechanical treatment Mineral nutrient regime Humidity regime Non-mineral nutrient regime Radiation (light, UV-B, X-ray) regime Rainfall regime Salt regime Watering regime Water temperature regime Standing water regime Pesticide regime pH regime Other perturbation	MIxS Plant-associated environmetal package [69] Yilmaz et al. [12]	XEO, XEML Environment Ontology [36] CRO, Crop Research Ontology [35]
Experimental design	Spatial coordinates Plant ID Plot ID Plot (x, y) coordinates Blocking Block ID Sub-block ID Sub-sub-block ID Superblock ID Row ID Column ID Other ID Replication Biological replication Technical replication Experimental unit		OBI, Ontology for Biomedical Investigations [66] STATO, Statistics Ontology [37] CRO, Crop Research Ontology [35]
Sample collection, processing, management	Plant body of interest (organ)* Plant product Organism count Sample temperature Oxygenation status of sample Sample salinity Sample storage duration Sample storage location Sample storage temperature Sampling time	CIMR [67]: Plant Biology Context Fiehn et al. [16]	
Observed variables	Phenotypic variables Trait* Method* Scale* Environmental variables Trait* Method* Scale* Data processing protocols	“Trait/Method/Scale” triplet approach applied by Generation Challenge Program, Crop Ontology [32] Shrestha et al. [33] Poorter et al. [26] Hanneman et al. [27]	PTO, Plant Trait Ontology [73] PO, Plant Ontology [74] CO, Crop Ontology [32] PATO, Phenotypic Quality Ontology [75] XEO, XEML Environment Ontology [36]

Attributes (concepts, subconcepts—in terms of ontology) marked by asterisk (*) are essential for a description of experiment (e.g. by Poorter et al. [26]); the rest forms an extended description. For some attributes possible values are listed (after colon)

The attributes from the “General metadata” section should allow to identify the research by providing some basic formal facts. First of all, an identifier of the dataset should be given, possibly a unified and permanent one. Additional important characteristics include a list of the contacts and other people involved, institutions, related projects and publications, data use policy, etc.

Another important aspect of research is to take note of the location and timing of an experiment. Depending on the nature of the study and scientific objectives, different initial time points might be crucial—sowing date or transfer date, treatment application time, etc. Duration of particular stages is also important. As regards location, certain amount of information about the experimental site should be provided for most types of research, in the form of a geographical identifier.

Plant material identification is a critical interoperability pivot and should receive careful attention when building a dataset. In the MI documents, a name “Biosource” has been coined for it. We recommend to define the biosource, i.e. biological object under study, by at least two attributes (as suggested by MIxS): one describing the organism’s species name, and the other the infraspecific name—either in the strict sense of McNeill et al. [29], or otherwise simply in the sense of the name of the plant accession, line, or variety, preferably included in a public collection of names, or in a namespace of an experimental station or a genebank (see also similar recommendations on the FAO/Bioversity Multi-Crop Passport Descriptors [30]). We also recommend indicating the source of the seeds for the experiment. Any additional descriptors, further specifying the biosource are optional, yet appreciated.

Owing to the central influence of environmental conditions on the phenotypic expression, accurate reporting on the conditions in which an experiment is performed is critical and warrants the level of details of the section “Environment” of the MIAPPE recommendations. It is our proposition to follow here Poorter et al. [26], who provided a table of attributes recommended to characterise the environment in which plant experiments are conducted. These recommendations encompass environmental descriptors for plants grown in growth chambers, greenhouses, and experimental fields and gardens. Collectively, they constitute a list of descriptors that should be used to describe basic properties of the experimental environment: aerial conditions, light, rooting conditions, fertilizing regimes, watering, and salinity.

Treatments are an inherent element of most phenotyping experiments. While it is impossible to list the types or names of all possible interventions that are used to test the reactions of plants, in MIAPPE’s section “Treatment” we provide some suggestions of experimental factors that should be added to the description, if applicable. Some of them are related to the environmental properties, whereas others are of artificial nature (e.g. mechanical treatment). With the help of this general list of treatments provided in MIAPPE, the description of the experiment should be completed with the details of all of the perturbations that appeared during the trial.

Plant phenotyping experiments are performed in a wide range of experimental designs. To obey the basic rules of replication and local control defined by Ronald A. Fisher, the (incomplete) block, row and column, or other layouts are used, both in the field and in greenhouse experiments. The description of the experimental design is an important part of metadata because any data analysis unaware of it cannot be valid. Especially, experimental units should be defined, i.e. “the groups of material to which a treatment is applied in a single trial” [31]; examples of the entities that play the role of experimental units in plants experiments are: single plant, a plot, or a pot (understood not as containers, but groups of plants).

Sample collection and processing information should include metadata related to phenotyping procedures, in particular sample collection protocol, sample preparation and treatments. If sampling is repeated in time, the time points must be specified.

A specific feature of phenotyping assays is the wide spectrum of observed variables and protocols (methods) used for measurements. This is reflected in MIAPPE in the section “Observed Variables”. Following the approach of the Crop Ontology platform [32, 33], we propose to describe the observed variables by three basic attributes: trait name, method, and scale. In this section, in addition to phenotypic variables (any plant characteristics that are measured in a phenotyping experiment), we also consider environmental variables, i.e. any attributes of the environment in which the phenotypic variables are recorded. Such variables are defined here because it is frequently necessary to measure various characteristics influencing the phenotype (potential covariates), possibly (or even usually) not just once, but periodically during the course of the experiment. Indeed, in the limiting situation one can imagine an assay in which the only variables measured are of the environmental type.

We are fully aware that MIAPPE suggests a description of the experiment that is rather extended in comparison to current practices. Hence, although we think that all of the attributes in Table 1 are needed to adequately describe each dataset, we accept that, in practice, the full complement of information may not be possible to collect, or might be unavailable to the person building the dataset. Therefore, we have selected and marked those descriptors deemed absolutely essential. These are also the attributes that we have used as defaults for constructing practical configurations and templates for data formatting (see “Metadata formatting” below). The rest of the attributes form an extended description.

### Annotation

Without proper semantic annotation, the wording used to name particular metadata elements might remain obscure. Referencing publicly available dictionaries and ontologies clarifies the concepts involved in the description, and should be done wherever possible. Ideally, the semantic layer present in an experiment’s description should also enable its use by automatic analysis and reasoning tools. In Table 1 we recommend ontologies for use in metadata annotation.

The selection of ontologies is based on [1] and on recent developments in this area. In addition to the reference ontologies for plants recommended by the Planteome project [34], e.g. Plant Trait Ontology (PTO), Plant Ontology (PO), ontology of phenotypic qualities (PATO), widely recognized and already frequently used vocabularies like Ontology for Biomedical Investigations (OBI), Gazetteer (GAZ), Environment Ontology (ENVO), NCBI Taxonomy, EURISCO catalogue, and species-specific ontologies developed as part of the Crop Ontology project, we recommend using the recently constructed:

- Crop Research Ontology [35]—especially for the MIAPPE sections General metadata, Environment, Treatments, and Experimental Design,
- XEO, XEML Environment Ontology [36]—especially for the section Environment and for environmental variables,
- STATO, Statistics Ontology [37]—for the section Experimental design and for unambiguously describing key statistical measures, such as p value, mean, standard deviation.

### Metadata formatting

As a sustainable exchange format for describing phenotyping experiments, we use the ISA-Tab, “Investigation-Study-Assay” format [22]. To facilitate formatting of MIAPPE-compliant datasets, we designed a novel ISA-Tab Phenotyping Configuration that satisfies the recommendations of the Minimum Information document.

**Fig. 1:**
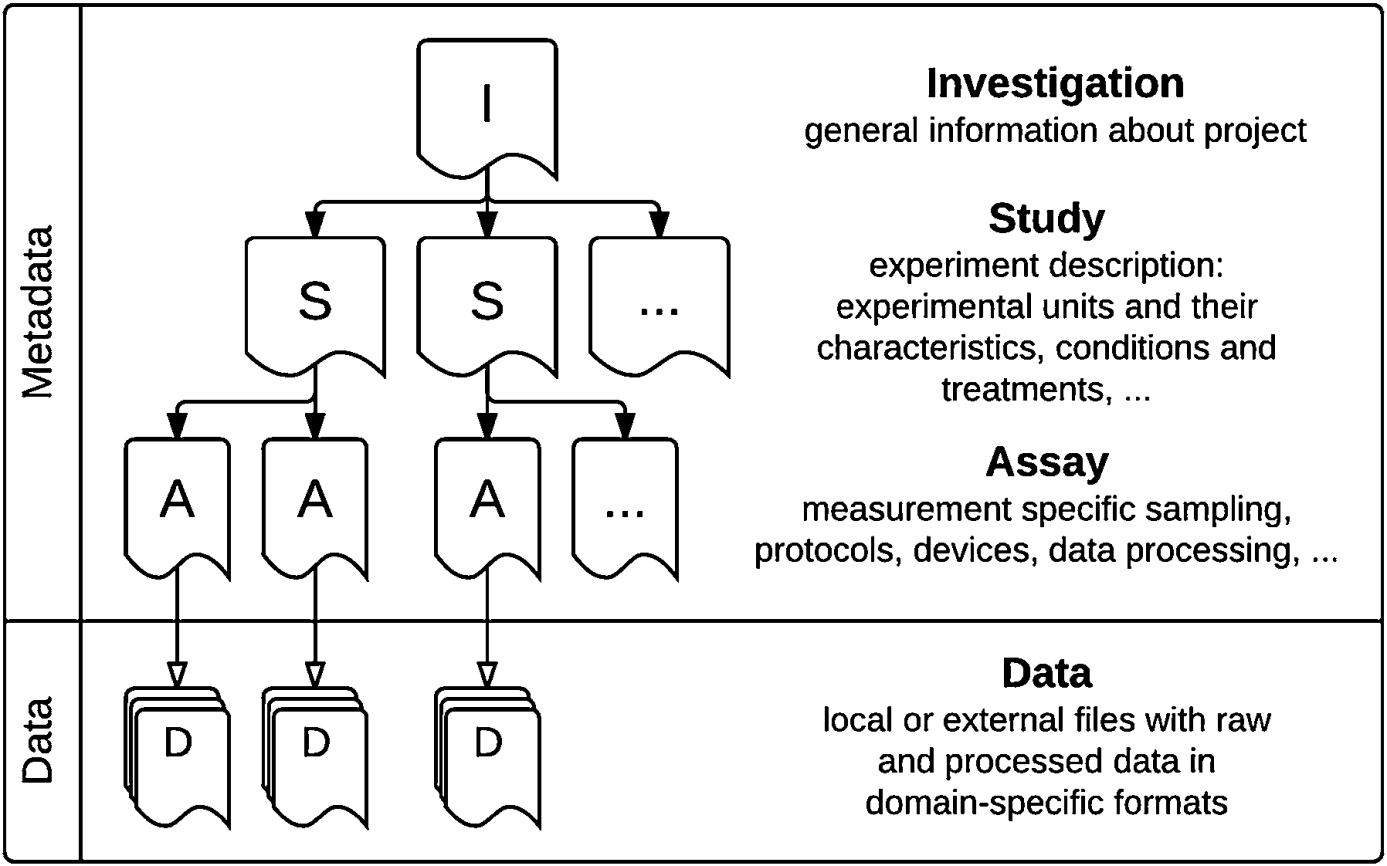
The structure of an ISA-Tab dataset

ISA-Tab is a general-purpose format to handle experimental metadata description. It consists of a set of tab-delimited text files, namely Investigation, Study, and Assay files, that are linked to each other to form a hierarchy, and describe different properties of a scientific undertaking (Fig. 1). In each dataset a sole Investigation file contains formal general information, e.g. the title, goals, methods, participants, etc. It also lists and formally describes one or more studies performed as parts of that undertaking. Each Study file represents a practical experiment, i.e. it describes the biosources (biological objects), experimental design, environmental conditions and treatments. An Assay file accommodates information about measurements, including description of samples collected from an experiment described in the Study for specific type of analysis, in particular their characteristics, processing and measuring procedures. The actual results of the measurements (or quantities derived from them—statistics) are contained in separate data files and linked to the corresponding metadata through a reference in the Assay file. There can be multiple Assay files per Study, each of them dedicated to a different assay type.

The Study and Assay files consist of columns describing properties of the objects under study; the objects are defined in rows. The allowed types of objects’ properties and the rules of their arrangements are defined in the ISA-Tab format specification [38]. Among the columns in Study and Assay files the main ones are so called “data nodes” (identifiers of groups of objects, objects, their parts, or samples taken from them; e.g. *Source Name*, *Sample Name*, *Extract Name*, *Assay Name*) that represent consecutive stages of the experiment. They are described by *Characteristics* (providing detailed object characterisation), *Factors* (naming experimental factors and their levels applied to each object), *Protocols* with *Parameters* (describing conditions and handling of the objects between particular stages), and *Comments* (any other unclassified content). All properties can be accompanied by their semantic annotation in dedicated fields (*Term Source REF* and *Term Accession Number* columns following the property column). *Raw Data File* and *Derived Data File* columns contain references to files in which raw and processed results of measurements are stored.

ISA-Tab configurations are extensions of the general specification, and provide additional requirements for types and arrangement of properties for particular purposes. Configurations can also be used to convey formatting to tools and services dealing with ISA-Tab files.

We propose a Phenotyping Configuration which facilitates formatting of MIAPPE-compliant ISA-Tab datasets. Within the configuration we define a dedicated Study file which provides a backbone for detailed description of field and greenhouse plant experiments, and a new type of Assay, a Phenotyping Assay, which deals with the information about phenotypic trait measuring procedures. The phenotyping Study files are compatible with other ISA-Tab Assays, so they can be useful for describing any plant experiment in which the environmental conditions are worth recording, irrespective of the types of measurements performed. The Phenotyping Assay can also be used with the default ISA-Tab configuration, and thus integrated in complex, multi-assay datasets that combine ISA-Tab-formatted results of diverse aspects of the analysed phenomena.

### MIAPPE to ISA-Tab mapping

The application of the format for phenotypic datasets consists in defining an ISA-Tab structure that serves as a container for MIAPPE concepts. This structure is defined in an XML file called ISA-Tab configuration. When preparing an ISA-Tab configuration for plant phenotyping, we had to allow for differences that occur between particular types of plant experiments, e.g. performed in different growth facilities. This is reflected in a varying set of attributes recommended in MIAPPE. Therefore, we propose an ISA-Tab Phenotyping Configuration that consists of a standard Investigation file, a Phenotyping Assay (described later) and three versions of a Study file:

- Basic Study—a general ordering of plant experiment specific metadata. It is a default initial description of all plant experiments, and needs to be extended by adding recommended MIAPPE attributes that are applicable in particular cases. In practice, it can be also used when very little is known about the origin of observations, e.g. for simple, external or legacy phenotypic datasets that should be formatted as ISA-Tab, without the ambition to satisfy the MIAPPE recommendations.
- Field Study/Greenhouse Study—extensions of the basic plant Study, featuring specific attributes for growth facilities and environmental information. They satisfy the MIAPPE requirements in terms of the most essential experiment attributes, yet should be further extended to include specific experimental factors present in the trial, and all of the other applicable recommended attributes that can be captured.

The three versions of plant Study use one common Phenotyping Assay file that describes phenotyping procedures and observed variables.

In Table 2 we describe the proposed ISA-Tab Phenotyping Configuration by showing how the MIAPPE attributes are mapped to the ISA-Tab elements in different plant Studies and in the Phenotyping Assay, and demonstrate how the description of the environment is included in field and greenhouse extensions through adding a number of protocols. A comparison of those protocols is shown in Table 3.

**Table 2:** Mapping of essential MIAPPE attributes to ISA-Tab structures in Phenotyping Configuration: Basic, Field and Greenhouse

Checklist section	ISA-Tab level	Checklist attribute	ISA-Tab structure	Basic	Field	Greenhouse
General metadata	Investigation	Unique identifier		●	●	●
		Title		●	●	●
		Description		●	●	●
Timing and location	Study	Timing Start of experiment (date) Duration (days/months/year)	Characteristics	●	●	●
		Experiment location Geographic location		●	●	●
Biosource	Study	Organism (taxon)	Characteristics	●	●	●
		Infraspecific name		●	●	●
		Seed origin		●	●	●
Environment	Study	Growth facility (growth chamber, GC/greenhouse, GH/open top chamber, OTC/experimental garden/field)	Characteristics	●	●	●
		Aerial conditions Air humidity (moisture) Daily photon flux (light intensity) Temperature (°C): Average day temperature Average night temperature	Protocol “Aerial conditions” with parameters		●	●
		Rooting conditions Rooting medium: aeroponics/hydroponics (water-based, solid-media based)/soil type (sand, peat, clay, mixed, etc.) pH For field: Plot size Sowing density For greenhouse: Container type Container volume Container dimensions Number of plants per container	Protocol “Rooting” with parameters		●	●
		Nutrients For soil: Extractable N content per unit ground area before fertiliser added Type and amount of fertiliser added,	Protocol “Nutrition” with parameters		●	●
		Watering For soil: Range in water potential (MPa) Irrigation from top/bottom/drip irrigation	Protocol “Watering” with parameters		●	●
Treatments	Study or Assay	All interventions being part of the experiment	Factor or Protocol with parameters		□	□
Experimental design	Study	Experimental units and their grouping (into blocks, superblocks etc.)	Characteristics, Factor, Protocol “Sampling” with parameters		●	●
Sample collection, processing, management	Assay	Plant body of interest (organ)	Characteristics	●	●	●
Observational variables	Assay	Phenotypic variables Trait Method Scale		●	●	●
		Environmental variables Trait Method Scale		□	□	□
Observations	Assay	Raw data	Raw data file	□	□	□
		Derived data	Derived data file	●	●	●

●—included in the ISA-Tab configuration; □—not included in the configuration, specific per experiment

**Table 3:** Comparison of default fields in the Study file in Basic, Field and Greenhouse ISA-Tab configurations

Basic	Field	Greenhouse
Source Name	Source Name	Source Name
Characteristics[Organism]	Characteristics[Organism]	Characteristics[Organism]
Characteristics[Infraspecific name]	Characteristics[Infraspecific name]	Characteristics[Infraspecific name]
Characteristics[Seed origin]	Characteristics[Seed origin]	Characteristics[Seed origin]
Characteristics[Study start]	Characteristics[Study start]	Characteristics[Study start]
Characteristics[Study duration]	Characteristics[Study duration]	Characteristics[Study duration]
Characteristics[Growth facility]	Characteristics[Growth facility]	Characteristics[Growth facility]
Characteristics[Geographic location]	Characteristics[Geographic location]	Characteristics[Geographic location]
	Protocol REF[Rooting]	Protocol REF[Rooting]
	Parameter Value[Rooting medium]	Parameter Value[Rooting medium]
		Parameter Value[Container type]
		Parameter Value[Container volume]
	Parameter Value[Plot size]	Parameter Value[Container dimension]
	Unit	Unit
	Parameter Value[Sowing density]	Parameter Value[Number of plants per container]
	Parameter Value[pH]	Parameter Value[pH]
	Protocol REF[Aerial conditions]	Protocol REF[Aerial conditions]
	Parameter Value[Air humidity]	Parameter Value[Air humidity]
	Parameter Value[Daily photon flux]	Parameter Value[Daily photon flux]
	Parameter Value[Length of light period]	Parameter Value[Length of light period]
	Parameter Value[Day temperature]	Parameter Value[Day temperature]
	Parameter Value[Night temperature]	Parameter Value[Night temperature]
	Protocol REF[Nutrition]	Protocol REF[Nutrition]
	Parameter Value[N before fertilisation]	Parameter Value[N before fertilisation]
	Parameter Value[Type of fertiliser]	Parameter Value[Type of fertiliser]
	Parameter Value[Amount of fertiliser]	Parameter Value[Amount of fertiliser]
	Protocol REF[Watering]	Protocol REF[Watering]
	Parameter Value[Irrigation type]	Parameter Value[Irrigation type]
	Parameter Value[Volume]	Parameter Value[Volume]
	Parameter Value[Frequency]	Parameter Value[Frequency]
	Protocol REF[Sampling]	Protocol REF[Sampling]
	Parameter Value[Experimental unit]	Parameter Value[Experimental unit]
Sample Name	Sample Name	Sample Name

The ISA-Tab Phenotyping Configuration is available online via our record registered with the BioSharing community [39].

### Observed variables

The specificity of the Phenotyping Assay (among other ISA-Tab assays, see [40]) lies in the fact that it collects information about different phenotypic and environmental variables that can be measured using different methods. The description of those variables is contained in a separate dedicated file, so-called Trait Definition File, referenced in the Phenotyping Assay as a parameter *Trait Definition File* of “Data transformation” *Protocol*. This file is an extension of the ISA-Tab specification, similar to the one that has been used in the ISA-Tab metabolomic configuration (see [41]) to describe metabolites.

The Trait Definition File contains a table with rows corresponding to variables and columns corresponding to the appropriate MIAPPE attributes, describing the trait, method and scale. In particular, it consists of the following columns:

- *Variable ID*—a local unique identifier of a variable, e.g. a short name, that is a key linking the definitions of variables with observations in Derived Data File,
- *Trait*—a name of the trait mapped to an external ontology; if there is no exact mapping, an informative description of the trait,
- *Method*—a name of the measurement method mapped to an external ontology; if there is no exact mapping, an informative description of the measurement procedure,
- *Scale*—units of the measurement or a scale in which the observations are expressed; if possible, standard units and scales should be used and mapped to existing ontologies; in case of a non-standard scale a full explanation should be given.

### Data

The data (observations or their functions) are represented in ISA-Tab in separate files, contained within the dataset or external, and are referenced in the Assay file as *Raw Data File* or *Derived Data File* properties. Formatting of the data file is not governed by the ISA-Tab specification, yet some recommendations usually exist within particular communities. In our implementation of MIAPPE, we do not restrict the format of the raw data in any way; it can be any custom, platform- or device-specific format, including texts, images, binary data, etc. Similarly, we do not restrict the format of any file referred to as *Derived Data File*; however, we require that the format be fully described in the corresponding *Protocol* “Data transformation” (a field that should precede the data reference, and explain how it was derived from the raw data, or from the previous derived data). If there is no description, the *Derived Data File* should be a standard, plain tab-separated sample-by-variable matrix. Its first column should contain (in the simplest situation) values from the *Assay Name* column in the Assay file, and the rest of the columns provide values for all variables. The names of those columns should correspond to the values in the *Variable ID* column in the Trait Definition File (see above). So, a default derived data format is an “Assay Name × Variable” matrix of observations, that can be quantitative or qualitative.

An extension of the above rule governing the format of the *Derived Data File* is possible by using values from another “data node” column (e.g. *Source Name*, *Sample Name*, *Extract Name*, etc.) as unique identifiers of the rows in the table with the associated observations. Thus, we can provide separate data files with measurements taken for different observational units, e.g., morphological traits like ‘height’ and ‘number of leaves’ can be assigned to the whole plant, whereas physiological traits can be restricted to samples taken from particular leaf of a plant. Also conveying data aggregated over “data nodes” is possible in this way.

### Implementations

The developed standard as well as the solutions proposed in this paper were first applied by the project partners dealing with phenotypic data. The main implementations, demonstrating possible approaches to follow the specification, are described below.

#### BII database at IPG PAS

At the Institute of Plant Genetics PAS, a BII database serving as an ISA-Tab-compliant storage for phenotypic data compatible with the MIAPPE standards has been launched. The BII software is part of the ISA Software Suite [28]. It consists of BII-Manager application which is used to validate ISA-Tab formatted datasets and store information to the database backend, and of BII Web application that provides a database front-end accessible via an Internet browser. The installation runs on a server at Poznań Supercomputing and Networking Center and is publicly available [42]. The system serves as a proof of concept and an illustration of the application of a generic, out-of-the-box tool for the basic needs of plant phenotypic data management.

Upon submission of the ISA-Tab archive to the administrator, the software is used to validate the files against a suitable configuration. If the validation is successful, the files get stored, and selected metadata are parsed into the internal structures for indexing and search. The content of the database is accessible via the web interface. Datasets can be browsed online, searched for by selected metadata terms, filtered according to the organism name and assay properties, and downloaded as ISA-Tab archives. It is also possible to declare a dataset as private, so that it is stored in private sections of the database and is inaccessible for unauthorized users. In its present version, the BII software cannot be used to retrieve data filtered by all metadata, so it does not use the full potential of the ISA-Tab format.

#### GnpIS-Ephesis at INRA

GnpIS [43] is an information system that allows data discovery and mining of genomic, genetic and phenomic data for plants and their bioagressors. GnpIS-Ephesis [11] allows experimental phenomics data mining, additionally including extended phenotype, genotype and environmental data and metadata integration. It offers users the possibility of creating multi-trial datasets suitable for various analyses (G × E meta-analysis, GWAS, etc.). GnpIS can be used, for example, to retrieve all data for a given diversity panel across several years or locations, all observations of a given phenological variable over several years, or all the data of a specific scientific study or project. Furthermore, all GWAS and genetic data integrated in GnpIS can be linked to a GnpIS-Ephesis experiment, allowing a better traceability and data exploration.

GnpIS-Ephesis allows to dynamically build and export ISA-Tab datasets, which demonstrates the capability of the format to handle results of diverse experiments, and to serve as a dataset exchange format. In the exported dataset the Investigation file represents the whole search results, and it integrates all the metadata, including the search parameters. There is one Study per trial. The Study contains only the subset of data corresponding to the user query with all the metadata necessary to ensure the reusability and the traceability of the data. The advantage of this implementation is that many public datasets are available through GnpIS, which allows to demonstrate the ISA-Tab format features.

An example the reader may look at is a dataset [44] that covers the winter wheat phenotypic observations from a French experimental network. It includes different traits (agronomic, quality, disease, phenology, etc.) measured at 10 experimental locations during 15 years (more than 700 trials) and for more than 1700 winter wheat genotypes [45], in the experimental network that allows to produce new varieties which can be registered to the French catalogue of varieties (CTPS) after their eight’s generation. Their identification is centralized by the French Wheat Genebank at Clermont Ferrand and is available through GnpIS. Several treatments were applied, like low fertilization, high nitrogen, etc. Each trial is stored as a single Study in ISA-Tab. Each Study lists the varieties used in a specific trial. The observation variables are collected in a dedicated ontology which is referenced in the ISA-Tab archive. Only derived data files are available.

#### Research data at IPK

IPK’s research data infrastructure comprises four layers [46]:

1. Primary research data: data generated manually or automatically in the course of experiments, derived data after post-processing of primary research data. Those data files are stored in IPK’s storage backend.
2. An in-house Laboratory Information Management System (LIMS), used for documentation of experimental metadata (experimental setup, used protocols etc.), based on primary data from layer 1.
3. Dedicated web-based information systems and databases, which provide access to curated and relationally structured data from layer 1, and which optionally link to the information from the LIMS (layer 2) [47].
4. The e!DAL data publication infrastructure [48], which provides DOIs for layer 1 data (especially datasets which are not covered by databases of layer 3), and which enables the public download of these datasets and registration of related technical metadata in the DataCite repository.

The ISA-Tab-based exchange format for plant phenotyping data was discussed among the collaborators from the German Plant Phenotyping Network (DPPN), the German Network for Bioinformatics Infrastructure (de.NBI), and partners from the European transPLANT project. Its application for future exchange of phenotypic data was agreed among partners from DPPN (especially IPK, *German Research Center for Environmental Health*, HMGU, Munich and *Research Center Jülich GmbH*, FZJ). It will serve as an exchange format for the semantic description of published data.

As an initial step, a reference experiment comprising multiple data domains was described using ISA-Tab structure and published [49] as a part of a research article of Junker et al. [50]. This dataset combines results and metadata from metabolite profiling, high throughput automated imaging and image analysis, as well as manual phenotypic measurements. All semantic and technical documentations, measured parameters, protocols and references to ontologies were manually described using ISA-Tab format. All raw files of such ISA-Tab formatted data publications are stored in the Plant Genomics and Phenomics Data Repository (PGP [51]), hosted at IPK using *e!DAL* as software infrastructure [52]. Recently IPK has published the first MIAPPE-compliant ISA-Tab container describing a high throughput plant phenotyping experiment including metadata, raw and processed images, extracted phenotypic features and manual validation data ([53], also stored in the PGP repository) as a data descriptor accepted at Nature’s Scientific Data journal [54]. The ISA-Tab files were manually filled and will be used as templates for the automated export of respective standardized metadata files describing all future high throughput plant phenotyping experiments. This dataset is shortly described as Dataset III in Discussion below.

#### GWA-Portal at GMI

GWA-Portal [55] is a web-application that allows researchers to upload their phenotypes and easily carry out Genome Wide Association Studies (GWAS) without installing any software. The GWAS results as well as the phenotypes can be shared with collaborators. By storing information ranging from phenotypes, germplasm to GWAS results in a single database, a comprehensive genotype-phenotype map can be constructed and thus allows researchers to do meta-analysis of pleiotropy. The development of GWA-Portal started before the MIAPPE was formulated and relies on the Genomic Diversity and Phenotype Data Model (GDPDM [56]) that was originally developed by Terry Casstevens from Ed Buckler’s lab. Although GDPDM was primarily designed for maize, it is not plant specific. In fact, the GWA-Portal instance that is hosted at the GMI, is used by the Arabidopsis community for storing phenotypes of the model plant *A. thaliana*. Initially GWA-Portal allowed the user to upload and download phenotypes as simple comma separated files. In the course of the transPLANT project the functionality was extended to support the ISA-Tab format. As GDPDM stores less information about phenotypes than what is defined in MIAPPE, we use the basic phenotyping configuration. Phenotypes in GDPDM are always stored as part of a study. This hierarchical structure maps quite well to the Investigation-Study-Assay set of the ISA-Tab format, with a study in GWA-Portal being equivalent to an investigation in ISA-Tab. As a result the mapping is quite straightforward.

The export functionality was implemented first. In order to avoid re-inventing the wheel, we tried to leverage the ISA-Tab toolchain and libraries as much of as possible. Specifically we used the ISAcreator library [57]. The import functionality was implemented shortly after. The ISAcreator library that we used for the export and import functionality is a GUI application and because we only use a small part of it, we suggested to the ISA-Tools team to create a dedicated lightweight library for parsing and creating ISA-Tab files.

## Discussion: best practices

### MIAPPE

MIAPPE recommendations provide a list of attributes that might be necessary to sufficiently describe a phenotypic dataset. One of its goals is to raise awareness of the researchers about the need to record a rich set of experimental metadata, especially environmental qualities which constitute a factor determining the phenotype in interaction with the genotype. Therefore, the MIAPPE requirements should serve as a checklist for the researchers recording the data to make them consider all aspects that might influence the experimental process and take note of those aspects. We suggest that the MIAPPE recommendations should be used in phenotyping projects already at the data management planning stage and be implemented according to the plan at all later stages of data collection.

We have selected a subset of MIAPPE attributes that seem common to the basic plant phenotyping cases, and marked them as obligatory ones. They should always be provided by the data producers to ensure some minimum standardisation in terms of data content. Inclusion of other attributes depends on the type of particular research, and it is up to the data owner to collect and describe all the factors in a responsible way, so that the dataset is correctly interpretable.

Selection of obligatory attributes raises the question of acceptance of the datasets by repositories. This is a community-wide issue. Repositories may wish to first flag submissions which are syntactically valid (a bare minimum for interoperation). Then, repositories may wish to insist on compliance with MIAPPE guidelines because there is an obvious long term benefit in terms of reuse, related to the notion of making data FAIR, i.e. Findable, Accessible, Interoperable and Reusable [58].

### ISA-Tab

Application of ISA-Tab format for plant phenotyping can be seen as a reference implementation of MIAPPE requirements. The textual and tabular nature of this format makes it usable for everyone without any dedicated tools or skills. We recommend using ISA-Tab as a format for experimental metadata collection and exchange. Whether to use the format to also store the datasets internally is a matter of individual decisions, based on existing solutions and needs.

The ISA-Tab Phenotyping Configuration contains the basic common subset of attributes that are necessary to describe a phenotyping experiment according to MIAPPE requirements. We propose using the configuration to ensure consistency of the phenotyping datasets formatted as ISA-Tab. Preparation of each dataset should involve providing all of the attributes named in the configuration, as well as identifying and adding to the dataset all other qualities present in the experiment (e.g. experimental factors and treatments, or supplementary protocols) as additional columns (e.g. *Factor*, *Characteristics* or *Protocol REF* and *Protocol Parameter*). Preparation of the ISA-Tab files can be done in three ways:

- manually in a text editor, adhering to the rules of the ISA-Tab format specification and Phenotyping Configuration; practically, the easiest way for the researchers recording the data might be to fill in a template (an empty dataset) provided by a data manager who prepares it based on the suitable Phenotyping Configuration through extending it by all adequate MIAPPE attributes and distributes it among the researchers;
- partly-manually, by using the ISA-Creator tool from official ISA software suite distribution with the Phenotyping Configuration to fill in and annotate experimental metadata;
- automatically, by preparing own scripts, possibly using the existing APIs, to construct ISA-Tab datasets based on manual data input (e.g. in GUI) or export from phenotypic databases.

Validation of the completed datasets against the rules provided in the ISA-Tab format specification and in the configuration can be done automatically by dedicated tools, e.g. ISA Validator.

In individual cases where adding the same new qualities for a number of experiments is necessary, we suggest creating a new local configuration based on the Phenotyping Configuration through extending it by the missing attributes, which will ensure the same structure for all of the experiments. It is important that the names of fields inherited from the original Phenotyping Configuration should not be changed in such derived configurations, and no fields should be removed, even if not used.

Similarly, the definition of Phenotyping Assay that we propose can be used as a starting point for building more specific extensions to the Phenotyping Configuration that would be appropriate for other common phenotyping measurements. For example, a high-throughput phenotyping protocol could be handled by an extension to the Phenotyping Assay, which should involve additional attributes defining phenotyping-facility-specific settings. Such extensions for the popular phenotyping platforms could be published, and included in the Phenotyping Configuration.

ISA-Tab is a very general format, suitable for a structured description of different kinds of experiments. The Investigation-Study-Assay model may look complicated at first; however, this very structure makes the format adjustable to various types of studies, and serves as a method of normalizing the metadata. Accepting a standard universal structure should remove the burden of learning new metadata arrangement formats every time a different dataset is produced. In the Phenotyping Configuration, we propose a data arrangement that should be applicable to the vast majority of plant experiments and phenotyping procedures, and which permits a straightforward integration with different assay types.

How to use ISA-Tab? Imagine a situation in which a collection of seeds of a number of crop varieties is given to researchers at different sites to compare the influence of the local environment on yield. They perform separate trials on, assumingly, the same set of objects, in similar—but not exactly the same—experimental designs. All general information about Biosource, Environment, Treatments and Experimental Design is to be given in separate Study files for each site. Data can later be aggregated across locations according to the obligatory attribute “Geographic location”. Imagine another situation, where an experiment is performed in one location, and many different researchers take samples from it, taking note of the identifier of the plant they analyse. In such a case, there is just one Study file, and a number of Assays for the individual researchers to record detailed description of handling of the samples and measurements.

We discuss the application of the presented approach by three examples of formatted datasets.

### Dataset I

Data contained in ‘dataset_basic_GMI_Atwell’ (Additional file 1) comes from the investigation described by Atwell et al. [59], and concerns *Arabidopsis* accessions. The data was downloaded in the ISA-Tab format from GWA-Portal at GMI. It has been formatted according to the basic phenotyping configuration. The Study file “s_Study1.txt” lists all the Biosources, i.e. *Arabidopsis* accessions, which are annotated by their identifiers in the GMI’s accession list. There are multiple replications of each accession; each one is assigned a unique Sample Name. The Sample Names are repeated in the Assay file “a_study1.txt” which links them to the rows of the Derived Data File “d_data.txt” through *Assay Name* column. The columns of the Derived Data File correspond to the 107 phenotypic variables stored on the GWAS platform and defined in the Trait Definition File named “tdf.txt”.

This example illustrates a situation in which the structure of the ISA-Tab archive does not reflect any actual experiment; the data, exported from an intermediary database, are in fact detached from most of their original metadata. Therefore, the information that is to be conveyed is very simple. One may say that in this situation the ISA-Tab structure, even in its basic configuration, is too complicated. However, obeying the rules even for simple datasets enhances greatly their interoperability.

### Dataset II

Data contained in ‘dataset_field_IPGPAS_Polapgen’ (Additional file 2) were obtained in a project aimed at studying reaction to drought in populations of barley recombinant inbred lines (RIL) [60]. The GeH population, obtained from a cross between Georgie and Harmal, consisting of 100 lines, was observed in a two-year field experiment in 2012 and 2013. The RILs and their parental forms constitute 102 biosources defined at the Study level in two files “s_study1.txt” and “s_study2.txt”, corresponding to the two years. The most important environmental data concerning soil type, field size, sowing density, and day temperature are provided as values of *Parameters* of the appropriate *Protocols*. Some information required by MIAPPE was not available, therefore a few columns in both Study files are empty. The phenotyping done on samples taken from field experiments is described in two Phenotyping Assay files, “a_study1_phenotyping_field2012.txt” and “a_study1_phenotyping_field2013.txt”. In the experiment eight phenotypic traits were measured; these are named and annotated in the Trait Definition File “tdf_polapgen_field.txt”. Additionally, two environmental variables were recorded: “water vapor pressure deficit” and “total precipitation”; they are also described in the Trait Definition File. The observations of phenotypic traits and of environmental variables are contained in data files “d_polapgen_field2012.txt” and “d_polapgen_field2013.txt”, corresponding to the two assays.

The GeH RIL dataset represents a very common case of a multi-environment study made with the same set of plant accessions. We decided to take the two environments—years—as two separate Studies; data are distinguishable upon processing by the value of the *Characteristics[Study start]* attribute. Another approach to handle different environments would consist in describing them within one Study file. In our case, however, the separation based on time-depended attribute seemed more convenient for data collection and the management of a whole series of experiments. In general, time points of sampling or data collection can be specified as a *Factor* or *Characteristic*.

Values of the environmental variables are constant over assays, as they represent the mean for the whole experimentation period and the whole experimental field. The same structure would hold single per-plot measurements. An environmental variable measured many times per experimental unit can be handled by splitting into a number of separate variables for each time point. Another approach would be to define a *Factor* “Time” and use it to define individual *Assay Name* for combinations of experimental units and time. Yet another solution would be to define a separate Assay to keep measurements of environmental variables.

### Dataset III

The experiment described and data contained in [53] have been acquired in the frame of a series of validation experiments in IPK’s high throughput plant phenotyping system for small plants. It assessed the effect of plant rotation during imaging (*Factor* “rotating”/”stationary”) as well as of soil covers (*Factor* “covered”/”uncovered”) on growth and development of 484 Arabidopsis plants. The dataset contains raw and processed images, extracted phenotypic features relevant for quantification of biomass (growth) and manual validation data. Detailed information about the experimental procedures and results can be found in [50]. The study has been described according to a MIAPPE-compliant ISA-Tab phenotyping configuration (Greenhouse Study) and was a part of data descriptor article [54]. The raw image files can be found in the “1135FA_images” folder. The subfolders are ordered and categorized into “camera_sensor” (vis/fluo/nir), “camera_view” (top/side) and “das” (day after sowing). The corresponding ISA-Tab files (Investigation, Study, and Assay files) for the semantical description are located in the “metadata” folder.

This dataset demonstrates the application of the ISA-Tab configuration (and MIAPPE) for a high throughput phenotyping experiment comprising time series measurements with different camera sensors. On the basis of this example the integration and representation of further related data (novel sensors, and importantly, environmental data) will be done at IPK.

## Conclusions

The results of research funded from public resources are expected to be publicly available, not only as a proof that the research was done, but also as a source of knowledge, or even input for further analyses. Open access to data is usually provided through open repositories (e.g. Dryad or Zenodo). They implement different policies of data formatting and description. Some accept objects (including datasets) of any type, assigning them simply an ID; others require adding a set of general attributes describing an object; some more ask for a specific data format. Repositories and databases of particular institutions and projects provide their own way for storage and access to data, most suitable for their needs, with an increasing policy toward Open Access. Future usability of datasets dispersed across all those repositories relies upon numerous factors: possibility to extract a specific dataset together with its metadata, comprehensible dataset formatting, completeness of its description, and clear meaning of individual elements of this description. Compatibility with other experimental results, also those of different types, is also not to be neglected in the context of data interoperability. Our work has been aimed at moving phenotyping data towards these objectives:

- The MIAPPE document, defining recommendations for phenotypic dataset description elements, helps to provide the right metadata in the dataset.
- The ISA-Tab format allows experimental metadata formatting, and thus inclusion of all important information within the dataset, making it exchangeable and independent of a data repository’s metadata policy. Flexibility of the format allows to export databases’ internal structures as ISA-Tab, while the definite rules for element arrangement make the experimental process traceable.
- The ISA-Tab Phenotyping Configuration provides mapping of MIAPPE requirements to ISA-Tab structures for the basic phenotyping situations, and thus facilitates dataset construction. Thanks to holding information on ontologies for particular attributes, it supports data annotation. A list of recommended ontologies for annotation of particular elements of experiment description assists in choosing formal terminology to clarify the wording, and thus avoiding ambiguity of the description. Ontological annotation is accommodated in ISA-Tab datasets.

The Minimum Information About a Plant Phenotyping Experiment document has been constructed as a result of consultations with a number of research groups within the transPLANT project and beyond, especially EPPN and DPPN. Although it is focused on classical phenotyping experiments, some attention in MIAPPE is also given to less frequently performed, but nonetheless important, experiments in aquatic and biotic conditions. Yet, a real application of MIAPPE in such situations would require more discussion with relevant practitioners. The same remark applies to observational studies.

Based on experimental data from high throughput plant phenotyping experiments at IPK using the LemnaTec platform, a first version of a high throughput phenotyping configuration has been prepared. This work builds the basis for a comprehensive plant phenomics experiment documentation and data publication pipeline. Indeed this kind of experiments comprising automated multisensor-imaging-based procedures can produce terabytes of data for each experiment. Handling such Big Data needs dedicated technologies and the level of resolution of related experimental metadata to be represented and published using ISA-Tab archives is still a matter of discussion. The selection of an adequate level of detail (geographical location of every single pot vs. location of the greenhouse), data volume (whether to remove low quality images or not) and processing stage (raw images vs. compressed/processed images) for data publication is linked to the technical capability of publication servers as well as institutional or journal policies. Nevertheless, the continuous documentation of the data lifecycle is a basic requirement for a consistent and seamless creation of ISA-Tab archives. We hope that the discussion with interested parties dealing with this type of experiments will allow a general or platform-specific High-Throughput Phenotyping Assay to be developed.

The textual nature of the ISA-Tab format makes it directly readable for everyone, without the need for any special software and support from computer scientists or bioinformaticians. Similarly, the construction of a dataset is possible manually, in a text or spreadsheet editor, by filling in a prepared template. A more advanced option is the preparation of an own implementation of data export/import as ISA-Tab based on the format specification to combine ISA-Tab with existing databases and tools. ISA-Tab is also supported by a free software suite, ISA-Tools, developed by ISA group [61] and members of the community. There are a number of tools and APIs for dataset construction, validation, analysis, management, and export to other formats. Certain functionalities of this official tools distribution are not yet provided, but the implementation of new user-friendly environments for dataset management is in progress [62, 63]. Further development of tools supporting formatting of data according to the given rules is an important step to promote adoption of the metadata standards.

Since the textual nature of ISA-Tab makes it not particularly convenient for automatic processing, the possibility to export ISA-Tab dataset structure to other formats is a useful feature. The existing tools provide, among others, JSON and RDF representations, as well as OWL for compatibility with the Linked Data. ISA-API [64] is going to further simplify programmatic approach to data formatting and management.

The ISA-Tab format has been accepted by the Nature Publishing Group for dataset publication, which additionally popularizes the format and encourages new users. More work is needed to achieve a widespread acceptance of the policy of data publication in the form of open resources. The FAIR Data Principles [58] that define the properties of a good dataset are a convenient remainder of the targets that are to be aimed at. Acceptance of the rules described in this paper will help to achieve these targets by the plant phenotyping community.

## Additional file

**Additional file 1.** An ISA-Tab-formatted dataset of Atwell et al. [59] from GMI.

**Additional file 2.** An ISA-Tab-formatted datasets of POLAPGEN-BD project from IPG PAS.

## Abbreviations

MIAPPE: Minimum Information About a Plant Phenotyping Experiment specification
ISA-Tab: investigation-study-assay tabular format for experimental metadata description
G × E: genotype by environment interaction
GWAS: genome-wide association study
